# A rare case of porphyria cutanea tarda in a patient with a homozygous hereditary hemochromatosis gene H63D mutation in the setting of hereditary hemochromatosis

**DOI:** 10.1016/j.jdcr.2024.05.023

**Published:** 2024-05-27

**Authors:** Jonathan Banta, Joshua Collins, Todd Kobayashi

**Affiliations:** a Department of Dermatology, Wilford Hall Ambulatory Surgical Center, Lackland AFB, San Antonio, Texas; b Department of Aerospace Medicine, 13th Fighter Squadron, Misawa AB, Misawa, Japan; c Department of Dermatology and Dermatopathology, Optum Medical Group, Colorado Springs, Colorado

**Keywords:** hereditary hemochromatosis, liver, porphyria cutanea tarda, skin

## Introduction

Porphyria Cutanea Tarda (PCT) is the most common cutaneous porphyria and manifests as photosensitivity, blistering, skin fragility, erosions, milia, and crusts on sun-exposed body areas.^1^, ^2^, ^3^ PCT is a hepatocutaneous disease of heme synthesis in which uroporphyrinogen decarboxylase (UROD) has reduced activity, resulting in the accumulation of porphyrins and heme intermediates in the liver and skin.^1^ Alcohol consumption, tobacco use, estrogen therapy, hepatitis C virus, and human immunodeficiency virus infections, iron overload, hereditary hemochromatosis (HH), and polychlorinated hydrocarbons are all established causes of PCT.^1^, ^2^, ^3^, ^4^ We present a case of PCT in a patient found to be homozygous for the hereditary hemochromatosis gene (HFE) H63D mutation in the setting of previously undiagnosed HH. Though the association between PCT and HH is well known, the association with the HFE H63D mutation, to our knowledge, has only been reported once in the medical literature.^5^

## Case report

**Fig 1:**
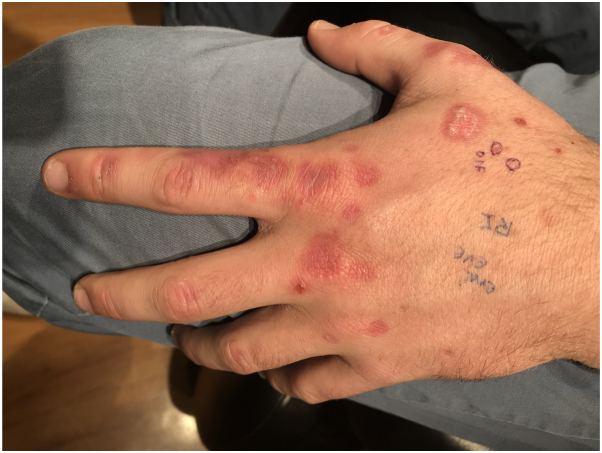
Numerous small noninflammatory vesicles are present on the patient’s left hand, along with hyperemic scars and some crusting on the dorsal fingers.

A 21-year-old Caucasian male was referred to dermatology for a 3-month history of recurring, asymptomatic blisters on his hands and fingers. He had no history of chronic conditions and did not take any daily medications. Physical examination revealed non-inflammatory vesicles on his dorsal hands with hyperemic scars and crusting on his dorsal fingers (Fig 1). The differential diagnosis included PCT, bullous impetigo, immunobullous disorder, and phytophotodermatitis.

**Fig 2:**
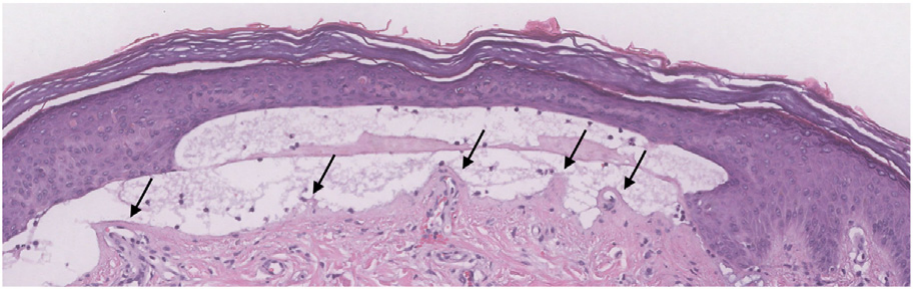
Histological findings from a punch biopsy showing a cell-poor subepidermal split affecting acral skin with festooning of the dermal papillae (*black arrows*) and perivascular hyaline cuffing of vessels within the dermal papillae (hematoyxlin and eosin, original magnification 20×).

**Fig 3:**
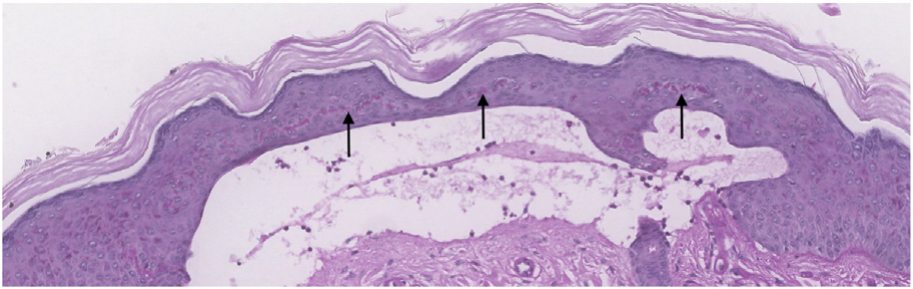
A periodic acid-Schiff stain displaying highlights to the perivascular cuffs, hyalinized “caterpillar bodies” within the epidermis (*black arrows*), and a thickened basement membrane (original magnification 20×).

A punch biopsy showed a cell-poor subepidermal split affecting acral skin with festooning of the dermal papillae and perivascular hyaline cuffing within the dermal papillae. Hyalinized material and dyskeratoic cells were noted in the overlying epidermis, forming “caterpillar bodies” (Fig 2). A periodic acid-Schiff stain highlighted the hyalinized perivascular cuffs in the papillary dermis, “caterpillar bodies” within the epidermis, and a thickened basement membrane (Fig 3). Uroporphyrins were elevated at 1093 μg/L, consistent with the diagnosis of PCT. Screening tests for hepatitis B, hepatitis C, and human immunodeficiency virus were negative. Given the patient's age and no other predisposing factors, genetic testing for HH was performed, and a homozygous H63D mutation in the HFE gene was found using polymerase chain reaction amplification followed by restriction enzyme digestion analysis.

The patient was advised to avoid alcohol and hepatotoxic medications and observe strict photoprotection. Initial blood work revealed a ferritin level of 312.2 ng/mL and a slight increase in aspartate aminotransferase (44 units/L) and alanine transaminase (73 units/L). He was prescribed fluocinonide 0.05% topical cream 2 to 3 times per day for flares. However, he noted no significant improvement in his disease. Phlebotomies were then done every 2 weeks until hemoglobin reached 11 g/dL or ferritin dropped below 20 ng/mL, resulting in complete remission of cutaneous disease.

## Discussion

PCT is either an acquired/sporadic (type I) or inherited (type II) disorder, with both types usually manifesting in the third or fourth decade of life. PCT (type I) is caused by extrinsic factors contributing to liver disease, such as alcohol consumption, tobacco use, estrogen therapy, hepatitis C virus and human immunodeficiency virus infection, iron overload, HH, and exposure to polychlorinated hydrocarbons, all of which result in decreased UROD activity restricted to the liver.^1^, ^2^, ^3^ In contrast, type II PCT is autosomal dominant with incomplete penetrance in which reduced levels of UROD activity of up to 50% can be found in the liver, red blood cells, and fibroblasts.^2^ Patients with PCT can expect to have a normal life expectancy when comorbid conditions are absent.^6^

PCT must be differentiated from other forms of cutaneous porphyria, as well as epidermolysis bullosa acquisita, hydroa vacciniforme, polymorphous light eruption, and phototoxic or photoallergic reactions. This can be achieved through a skin biopsy and measuring the excretion of urinary and stool porphyrins.^2^^,^^6^ Typically, skin biopsy shows a cell-poor subepidermal blister with periodic acid-Schiff-positive hyaline cuffing of papillary dermal vessels.^6^ Periodic acid-Schiff-positive amorphous hyaline material is often carried up into the epidermis forming “caterpillar bodies,” a distinct finding in PCT. Direct immunofluorescence shows IgG deposits within vessel walls of the papillary dermis.

In this case, iron overload in the setting of HH played a vital role in the development of PCT in an otherwise healthy individual. The role of iron in PCT is complex, and several hypotheses have been proposed regarding the pathogenesis of the disease. Hepatic iron overload is present in nearly all cases, and elevation in plasma iron is present in 50% of cases.^2^^,^^4^ It is theorized that iron may inhibit UROD through direct suppression or indirectly as an essential co-factor in generating the UROD inhibitor uroporphomethene.^2^^,^^4^^,^^6^ The benefit of removing plasma iron through phlebotomy supports the critical role of iron overload in this disease.^2^^,^^4^

HH is an autosomal recessive disorder and the most common genetic disease in populations of European descent.^7^ Mutations in the HFE gene located on chromosome 6p21.3, C282Y and H63D, are essential factors in the development of PCT.^2^^,^^3^^,^^6^^,^^7^ Studies have shown that homozygosity of C282Y mutation leads to earlier onset of symptoms in PCT.^2^^,^^4^ The H63D mutation has also been associated with PCT but is much less common. In 1 study, 15% of PCT patients carried the homozygous C282Y mutation, and 8% carried the homozygous H63D mutation.^8^ The H63D mutation alters the normal HFE product’s affinity for its ligand, the transferrin receptor, contributing to an iron overload state.^7^^,^^9^ In patients with HH, iron overload tends to be higher in those homozygous for the C282Y mutation when compared to those that are homozygous for the H63D mutation.^7^^,^^9^ In the case presented, the patient was found to be homozygous for the H63D mutation and negative for the C282Y mutation.

Although treatment of PCT varies depending upon the severity of the condition, photoprotection and avoidance of well-known triggers, such as alcohol and estrogens, is both prophylactic and therapeutic in most cases of PCT.^2^^,^^4^ Other mainstays of treatment include hydroxychloroquine, chloroquine, and phlebotomy.^10^ Our patient achieved complete remission through serial therapeutic phlebotomies every 2 weeks.

In conclusion, we report the clinical, histological, laboratory, and genetic work-up of a 21-year-old Caucasian male with PCT in the setting of previously undiagnosed HH caused by a homozygous H63D mutation in the HFE gene, which has rarely been reported.

## Conflicts of interest

None disclosed.
